# From genome to evolution: investigating type II methylotrophs using a pangenomic analysis

**DOI:** 10.1128/msystems.00248-24

**Published:** 2024-05-02

**Authors:** Dipayan Samanta, Shailabh Rauniyar, Priya Saxena, Rajesh K. Sani

**Affiliations:** 1 Department of Chemical and Biological Engineering, South Dakota School of Mines and Technology, Rapid City, South Dakota, USA; 2 BuG ReMeDEE Consortium, South Dakota School of Mines and Technology, Rapid City, South Dakota, USA; 3 2-Dimensional Materials for Biofilm Engineering, Science and Technology, South Dakota School of Mines and Technology, Rapid City, South Dakota, USA; 4 Data Driven Material Discovery Center for Bioengineering Innovation, South Dakota School of Mines and Technology, Rapid City, South Dakota, USA; University of California Irvine, Irvine, California, USA

**Keywords:** hypothetical proteins, isozymes, methane, persistent, PPanGOLLiN, serine pathway

## Abstract

A comprehensive pangenomic approach was employed to analyze the genomes of 75 type II methylotrophs spanning various genera. Our investigation revealed 256 exact core gene families shared by all 75 organisms, emphasizing their crucial role in the survival and adaptability of these organisms. Additionally, we predicted the functionality of 12 hypothetical proteins. The analysis unveiled a diverse array of genes associated with key metabolic pathways, including methane, serine, glyoxylate, and ethylmalonyl-CoA (EMC) metabolic pathways. While all selected organisms possessed essential genes for the serine pathway, *Methylooceanibacter marginalis* lacked serine hydroxymethyltransferase (SHMT), and *Methylobacterium variabile* exhibited both isozymes of SHMT, suggesting its potential to utilize a broader range of carbon sources. Notably, *Methylobrevis* sp. displayed a unique serine-glyoxylate transaminase isozyme not found in other organisms. Only nine organisms featured anaplerotic enzymes (isocitrate lyase and malate synthase) for the glyoxylate pathway, with the rest following the EMC pathway. *Methylovirgula* sp. 4MZ18 stood out by acquiring genes from both glyoxylate and EMC pathways, and *Methylocapsa* sp. S129 featured an A-form malate synthase, unlike the G-form found in the remaining organisms. Our findings also revealed distinct phylogenetic relationships and clustering patterns among type II methylotrophs, leading to the proposal of a separate genus for *Methylovirgula* sp. 4M-Z18 and *Methylocapsa* sp. S129. This pangenomic study unveils remarkable metabolic diversity, unique gene characteristics, and distinct clustering patterns of type II methylotrophs, providing valuable insights for future carbon sequestration and biotechnological applications.

**IMPORTANCE:**

Methylotrophs have played a significant role in methane-based product production for many years. However, a comprehensive investigation into the diverse genetic architectures across different genera of methylotrophs has been lacking. This study fills this knowledge gap by enhancing our understanding of core hypothetical proteins and unique enzymes involved in methane oxidation, serine, glyoxylate, and ethylmalonyl-CoA pathways. These findings provide a valuable reference for researchers working with other methylotrophic species. Furthermore, this study not only unveils distinctive gene characteristics and phylogenetic relationships but also suggests a reclassification for *Methylovirgula* sp. 4M-Z18 and *Methylocapsa* sp. S129 into separate genera due to their unique attributes within their respective genus. Leveraging the synergies among various methylotrophic organisms, the scientific community can potentially optimize metabolite production, increasing the yield of desired end products and overall productivity.

## INTRODUCTION

The field of genomics has predominantly focused on utilizing reference genomes as guiding maps, offering insights into the genetic makeup of a “typical” individual within each species (1). However, a single reference genome imposes limitations and fails to capture the full extent of genetic variation within a species. To address this issue, researchers have begun creating and utilizing pangenomes, which represent collections of all genomic DNA sequences identified across individuals within a species (2). The increase in the number of sequenced genomes has led to the recognition of graph-based pangenomes as a platform for studying diversity in a population or species, ranging from point mutations to large chromosomal rearrangements (3). Pangenomes can be represented as directed graphs, capturing structural and single variants (4). Despite their benefits, incorporating graph genomes into research practice is challenging due to the need for new tools, data structures, and formats as well as the difficulty in integrating them with existing software and databases (5). However, pangenomes have enabled notable discoveries that would have been difficult or impossible with traditional reference genomes and are evident from the identification of 51% gene families from the dispensable genomes of *Glycine soja* in 2014 (6).

Methanotrophs, a subset of methylotrophs, are crucial in regulating the global carbon cycle by converting methane into carbon dioxide (7). They are classified into three categories: type I and type X (γ-proteobacteria), type II (α-proteobacteria), and Verrucomicrobia (8). Type I and type II methanotrophs have distinct metabolic capabilities owing to differences in their intracellular structures, carbon assimilation pathways, and other metabolic features (9). Unlike type I methanotrophs, type II lacks intracytoplasmic membranes, which house methane monooxygenases (MMOs) (10). As a result, type II methanotrophs have cytoplasmic and transmembrane MMOs (11). Additionally, type I methanotrophs utilize the ribulose monophosphate pathway for carbon assimilation, while type II methanotrophs utilize the serine pathway (10, 12). This pathway enables type II methanotrophs to co-incorporate methane and CO_2_, giving them more versatility in carbon assimilation (13). Moreover, type II methylotrophs have the ability to fix atmospheric nitrogen, making them suitable for growth in nitrogen-limited environments (14). They also have a high acetyl-CoA flux, making them a potential microbial cell-factory platform for methane-derived biomanufacturing (13). Furthermore, through the application of pangenomic analysis, Oshkin et al. were able to distinguish two closely related type II methanotrophic genera, *Methylosinus* and *Methylocystis*. Their study revealed a diverse range of enzymes involved in methane oxidation and dinitrogen fixation, as well as genomic determinants for cell motility and photosynthesis within the accessory genome of these methanotrophic bacteria (15). Overall, the distinct metabolic features of type II methylotrophs make them a fascinating subject of study and a potential tool for methane biomanufacturing.

**Fig 1:**
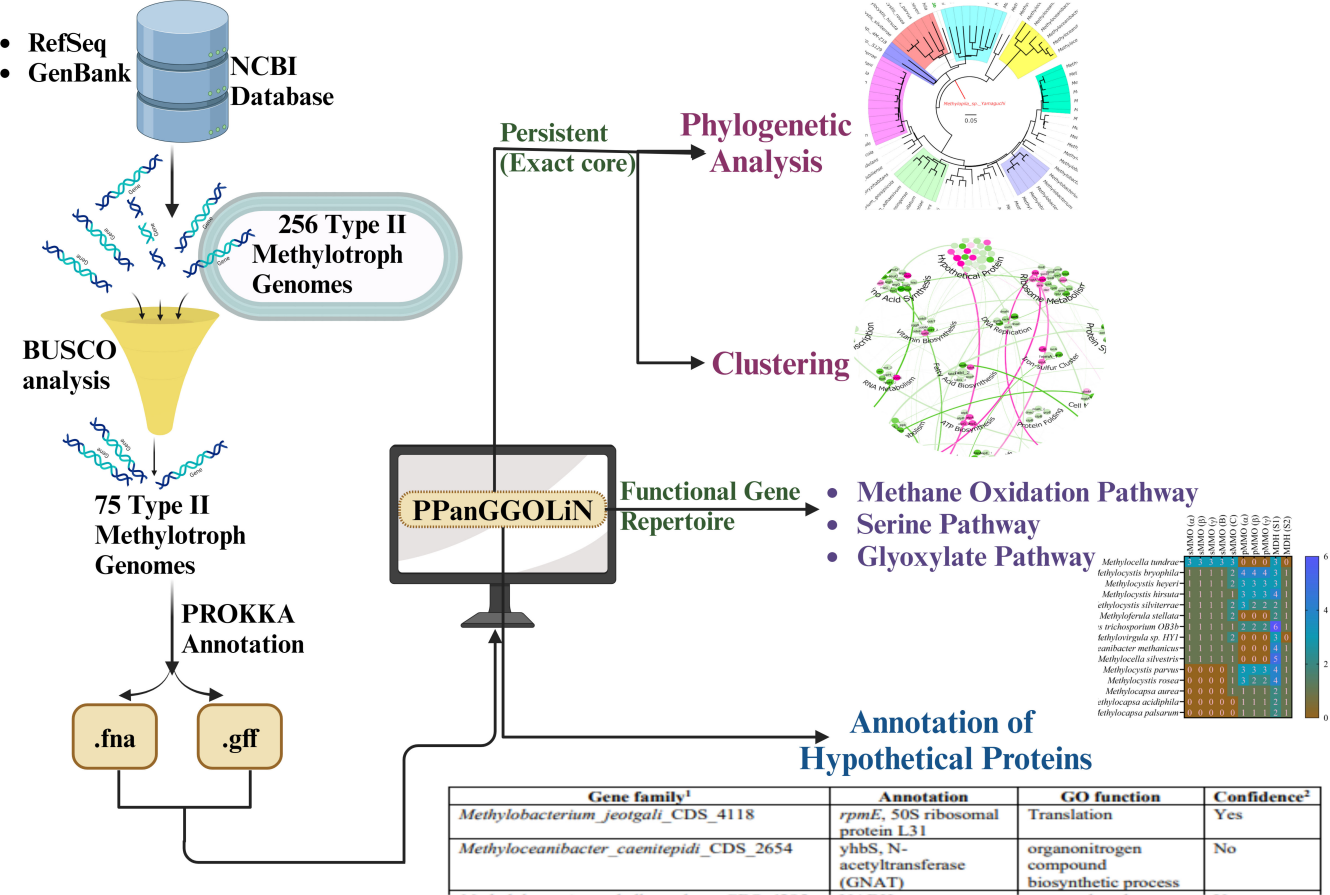
Schematic overview of the study determined to analyze the genetic pattern in type II methylotrophs using pangenome.

The primary objective of this study is to comprehensively explore the genetic diversity and evolutionary patterns of type II methylotrophs (Fig. 1). To achieve this, we employed a cutting-edge pangenomic approach to analyze the complete genetic content of type II methylotrophs in various environments. Through this comprehensive analysis, we aim to identify the unique genetic traits and pathways that underlie the evolutionary processes of type II methylotrophs. The findings of this study will have significant implications for developing biotechnological applications and strategies for mitigating methane emissions, thereby providing a valuable contribution to the field of environmental microbiology.

## MATERIALS AND METHODS

### Genome mining and selection

The genera and species of type II methylotrophs available in the National Center for Biotechnology Information (NCBI) Refseq and GenBank assembly database were compiled into a Microsoft Excel (v365) file (16, 17). The genomic fasta (.fna) files of these organisms were obtained from the NCBI Refseq and GenBank using an automated Python program that utilized file transfer protocol. The completeness of the selected genomes was assessed using Benchmarking Universal Single-Copy Orthologs (BUSCO) analysis, which calculated the percentage of completeness (C), fragmented (F), and missing genes (M) in each genome (18). Assembly statistics such as scaffold N50 and L50, contig N50 and L50, coverage, total gap length, and spanned gaps were also collected for each genome to ensure quality. Genome assemblies with at least 94% completeness, less than 1,000 total gap length, and less than 5 spanned gaps were selected for pangenome analysis.

### Genome annotation

To remove the biasness and variations in gene prediction and annotations, all the genomes used in pangenome analysis were annotated using Prokka v1.8 with *ab initio* algorithm (Prodigal v2.6.3) (19). To ensure accurate gene annotations, genus-specific parameters were employed. This approach allows the annotation algorithm to consider the same gene and codon patterns identified within the particular genus under study. Prokka employs an integration pipeline that incorporates various databases to enhance the annotation process. The databases used BLASTp, UniProt, RefSeq, Rfam, and TIGRFAMs, which provide valuable information for the identification and characterization of proteins. SignalP is used to identify signal peptides, aiding in the prediction of protein localization and its function. The prediction of transfer RNA (tRNA) and ribosomal RNA (rRNA) was performed using Aragorn (v1.2.41) and Barrnap v0.9, respectively. To identify CRISPR sequences, the software minced v.4.2 was utilized.

### Pangenome analysis

The pangenome analysis was performed using graph-based analysis tool PPanGGOLiN (20). PPanGGOLiN is a statistical and graphical model-based tool that can construct pangenomes for large sets of prokaryotic genomes. The input files consisted of PROKKA-annotated genomic FASTA (.fna) and GTF files, with their coding regions classified into homologous gene families. The PPanGGOLiN pipeline was uploaded onto Google Colab for pangenome analysis. The tool utilizes information on protein-coding genes and their genomic context to build a graph, where each node represents a gene family, and each edge indicates genetic contiguity between families that are neighbors in the genomes. This approach has the advantage of being able to handle fragmented genome assemblies and maintain linkages between gene families even when genome assembly gaps exist. Orthologous gene clustering is performed using a sequence similarity cutoff of 50% and sequence coverage cutoff of 80%. This ensures that genes with sufficient similarity and coverage are clustered together as orthologs. The output file contained the corresponding gene families from the pangenome, along with partition and quality parameters, such as percent identity (*p*_ident_), expectation value (*e*-value), and bit score for each input gene. PPanGGOLiN classified the genes into three categories: persistent, cloud, and shell partitions. The persistent gene families refer to the conserved gene families present across the entire bacterial genomes under study. The genes present in an intermediate number of genomes are labeled as shell, whereas the unique genes specific to a particular species are termed as cloud genes (21, 22).

### Physical protein-protein interaction and enrichment

To analyze the physical protein-protein interactions (PPIs) and subsequent gene ontology (GO) interactions between the persistent genes, the STRING (Search Tool for Interacting Genes Retrieval) database was utilized, which is a precomputed global resource for predicting functional associations between proteins (23). To enrich the PPI network, the persistent gene set was taken as input into the STRING database. The enriched genes with a *P*-value less than 1.6 × 10^−16^ were visualized using Cytoscape (version 3.9.1), a PPI visualization software (24). The Cytoscape plugin, StringApp, was used to perform pathway enrichment analysis and import PPI networks from the STRING database to Cytoscape. *Methylosinus trichosporium* OB3b was selected as the organism, and a confidence score cutoff of 0.40 was used to find the potential interactions between the genes. To unveil the relationships between the GO terms related to the input gene set, enrichment interactions were performed (25). The most enriched GO terms were screened based on the false discovery rate, which was less than 1.0 × 10^−6^. In the GO-enrichment network, the nodes corresponded to the GO terms, and the edges depicted the interactions. A heatmap was generated to show the relevance of the enriched GO terms (each node) in a particular molecular function (MF)/biological process (BP)/cellular component (CC).

The pangenome graph file generated by the PPanGGOLiN pipeline was visualized using Gephi software (version 0.9.2), enabling graph visualization and manipulation (26). The nodes and edges representing the exact core, which are shared by all the organisms, were selected and filtered. Statistical analysis was performed, encompassing metrics such as average degree, average weighted degree, network diameter, modularity, average clustering coefficient, and average path length. These analyses provide insights into the structure and characteristics of the pangenome network. To enhance visual comprehension, the nodes were ranked based on the number of sequences, and a color ranking was assigned to represent the modularity class. Initially, the “Noverlap” layout was employed for arranging the nodes, followed by manual grouping of nodes based on their biological functions. Functional categories for gene families were obtained from the UniProt database (27). These categories aid in understanding the functional diversity within the pangenome network. The final network image was downloaded as a vector image with default presets and subsequently edited using Inkscape software (version 1.2). The editing process involved marking the node clusters with their respective functional categories, thereby improving the clarity and interpretability of the network visualization.

### Gene ontology

The output generated by PPanGGOLiN resulted in a comprehensive matrix file containing gene families and their associated gene IDs specific to type II methylotrophs. Obtaining UniProtKB information for the exact core data was crucial; however, each gene family had multiple UniProtKB matches (28). Since each gene family represented a cluster of genes from at least the specified number of organisms, or more, it was expected to have multiple ID matches. Therefore, mapping the exact core gene families with gene ID data was necessary to obtain the complete gene list. The original genome annotation file in .gff format, obtained from Prokka, included the UniProtKB ID for each gene. To extract the UniProtKB information for each gene ID, a Python script was utilized, which extracted the relevant data from the Prokka annotation file. The extracted information was then consolidated into a single Excel (.xlsx) file. This resulting output, containing the UniProtKB IDs for the entire exact core gene list, was subsequently fed into the “Retrieve/ID mapping” tool provided by UniProt. This tool facilitated the retrieval of gene ontology information corresponding to the UniProtKB IDs, enriching the data set with valuable functional annotations associated with the genes. The GO terms associated with the gene families were retrieved and grouped, enabling the determination of the total gene family count and gene count for each specific GO category. To assign scores to each GO term, the percentage of genes within that category was calculated in relation to the total mapped genes. To visualize the distribution of GO terms across different categories such as BP, MF, and CC, plots were created using ggplot2 in Python v3.10.8.

### KEGG pathway analysis

Pathway analysis and functional categorization of gene families in this study were performed using the KEGG Mapper search tool (29). The Enzyme Commission numbers were used as references to search pathway IDs for the input enzyme data. To achieve functional categorization of gene families in the shell and cloud partitions, KEGG BlastKOALA (KEGG Orthology and Links Annotation) and GhostKOALA were utilized, respectively (30). The FASTA amino acid sequence file for both partitions was uploaded to the KEGG server, with the “genus_prokaryotes” and KEGG genes database selected for the search.

### Putative annotation to hypothetical proteins

The online webserver MOTIFSearch (https://www.genome.jp/tools/motif/) was employed to determine the motifs present in each hypothetical protein belonging to the exact core. This webserver offers a powerful platform for motif analysis by leveraging various databases and pattern repositories. This webserver leverages information from various sources including Pfam, NCBI-CDD (TIGRFAM, COG, and SMART), and PROSITE pattern to identify motifs within the amino acid sequences of the proteins. To ensure reliable results and minimize the false discovery rate, a statistical analysis was conducted using a low *e*-value threshold. The cutoff value of 1 × 10^−5^ was chosen as the criterion for significance. This threshold helps identify motifs with a high level of confidence, enhancing the accuracy and reliability of the motif predictions. Therefore, the putative functionality of the proteins was determined through a motif-based search, which helped assign potential functional annotations based on identified motifs. To ensure enhanced reliability and accuracy in determining the functionality of the identified genes, individual FASTA files were subjected to ProteInfer, a deep network for protein functional inference. By utilizing this server, we obtained GO terms along with associated confidence scores ranging from 0 to 1, providing a more comprehensive understanding of the functional annotations (31).

### Phylogenetic analysis

The PPanGGOLiN workflow utilized a command-line tool to generate alignment files (.aln) encompassing the entire core gene families. These alignment files were subsequently converted to FASTA format. The resulting FASTA alignment files were concatenated, and further evolutionary analyses were carried out using MEGA v11. To infer the evolutionary history, the neighbor-joining method was employed. The Jones-Taylor-Thornton (JTT) matrix-based method was utilized to compute the evolutionary distances, which are measured in the number of amino acid substitutions per site for the final concatenated sequence file. To enhance the reliability of the analysis, the method was bootstrapped with 1,000 replications. In addition to the phylogenetic tree, pairwise distances and an overall mean distance analysis were conducted. Ambiguous positions were eliminated for each sequence pair using pairwise deletion. The resulting tree was drawn to scale, with branch lengths corresponding to the evolutionary distances employed in inferring the phylogenetic relationships. Finally, the tree was exported to an offline tree editing software FigTree v1.4.4 for final quality processing, providing a visually appealing representation of the evolutionary relationships (32).

### Mapping of the genes based on pathways

A significant body of scientific literature exists that elucidates the genetic foundations of type II methylotrophs. To compile a comprehensive list of crucial pathway genes commonly found in type II methylotrophs, a meticulous manual search was conducted using PubMed and PubMed Central literature databases. In order to obtain the amino acid sequences corresponding to the identified genes, the UniProt Retrieve/ID mapping tool was employed, and the sequences were downloaded in the FASTA format. This FASTA file was subsequently uploaded to Google Collab (PPanGGOLiN), where the “align” command was utilized to align the gene set with the type II methylotrophs pangenome (33). The output data encompassed the matched gene families from the pangenome, along with partition and quality parameters such as percentage identity (*p*_ident_), expectation value (*e*-value), and bitscore for each input gene. The term “*p*_ident_” denotes the percentage of amino acid sequence positions that have identical residues, while “bitscore” is a statistical measure derived from a raw alignment score that assesses sequence similarity independently of sequence length and database size. “*E*-value” indicates the likelihood of sequence similarity occurring by chance. To generate a presence/absence matrix for the crucial genes, the corresponding gene family matrix data were retrieved. This matrix data were then utilized to create a heatmap using MS Excel v365.

## RESULTS

### Genome mining and genome screening for pangenomic studies

**Fig 2:**
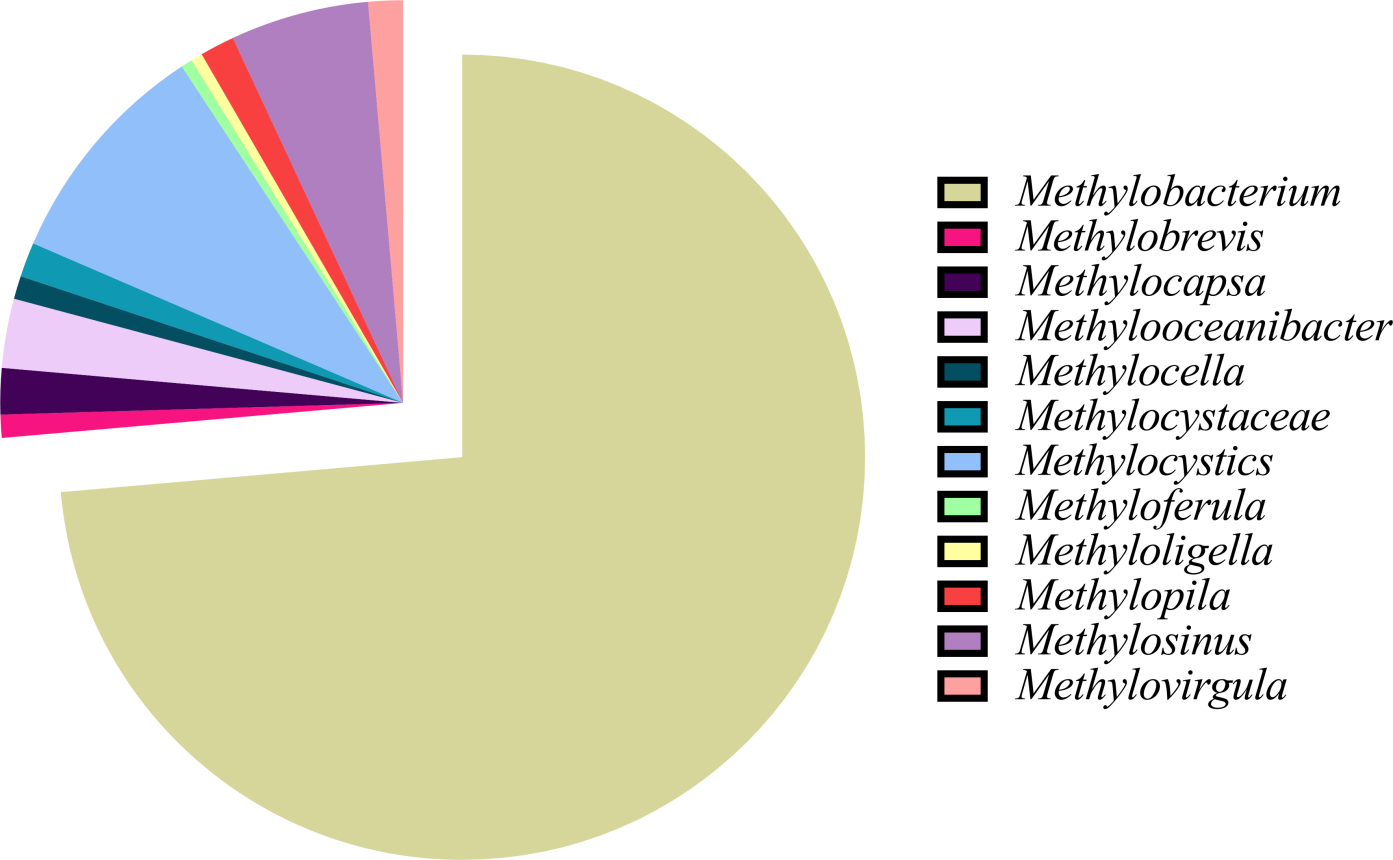
Taxonomic distribution of type II methylotrophs within respective genera.

We utilized the NCBI RefSeq and GenBank databases and collected 216 type II methylotrophs of various genera (34, 35). Figure 2 visually represents the frequency distribution of these organisms within their respective genera, offering valuable insights into their taxonomic distribution. The distribution analysis reveals a diverse array of 11 distinct genera that have been identified thus far within the type II methylotrophs. Notably, among these genera, there exists an unclassified genus that has been designated with the family name Methylocystaceae. The predominant genus within the type II methylotrophs is *Methylobacterium*, comprising a significant portion (73.6%) of the community.

In addition to curating the data set, we performed BUSCO analysis to assess the completeness of the genomes. The visualization in Fig. S1 provides a clear depiction of the screening process, highlighting the assessment of each genome’s completeness, identification of missing fragments, and evaluation of duplication levels. A genome with less than 94% completeness was classified as a poor-quality genome for this study. This criterion has been established to ensure the inclusion of at least two organisms from each genus, allowing for a representative sampling across the taxonomic groups. By evaluating each genome against this critical criterion, we ensured that only genomes meeting the required standards were selected for subsequent analysis. From the initial collection of 216 organisms, the BUSCO analysis successfully screened and retained 75 organisms that met the predefined criteria (≥94%) for genome completeness.

The complete data set comprising 75 genomes, including information on genome size, GC content, genome coverage, and isolation site, has been compiled and presented in Table S1. Notably, the range of genome sizes varied significantly, with the smallest genome size observed in *Methylooceanibacter marginalis* (2,997,425 bp), while the largest genome size belonged to *Methylobacterium nodulans* (8,839,022 bp). The GC content across the genomes ranged from 58.3% to 73.0%, with an average value of 67.5%. Among the 75 genomes, 44 genomes exhibited a GC% equal to or greater than the average, suggesting a predominance of GC-rich genomes within the type II methylotroph community. Regarding genome coverage, 42 genomes had coverage values exceeding 100×, with the highest coverage reaching 1,680×. Conversely, 26 genomes displayed coverage below 100×, with the minimum coverage value noted at 12× for a particular genome. It is important to mention that sequence coverage data were unavailable for seven genomes in the assembly statistical report. Moreover, genomes with substantial total gap length and spanned gap values were excluded from the current study. All 75 organisms were found to inhabit mesophilic environments, with temperatures ranging from 25°C to 32°C. This temperature range represents the typical habitat for these type II methylotrophs.

### Pangenome analysis and retrieval of precise core gene sets

A total of 256 precise core gene families were identified, comprising 20,188 genes distributed across 75 genomes. To establish a mapping between the 256 exact core gene families and their corresponding PROKKA gene IDs, a combination of Microsoft Excel and Python code was employed. Subsequently, the UniProt information associated with each PROKKA gene ID was retrieved from a consolidated Excel file, containing PROKKA annotations for all organisms examined in the study. These mappings allowed us to gather data on 241 distinct UniProtKBs, including their respective gene family affiliations and GO terms. It is noteworthy that each of the exact core gene families was represented in the final list of UniProtKB entries. For a comprehensive overview of the information pertaining to each specific UniProtKB, we refer readers to Table S2, which depicts an Excel file downloaded from UniProt, encompassing all relevant details associated with the respective UniProtKB entries. The functionality of the hypothetical proteins identified in this study has thoroughly been examined and discussed in subsequent sections.

**Fig 3:**
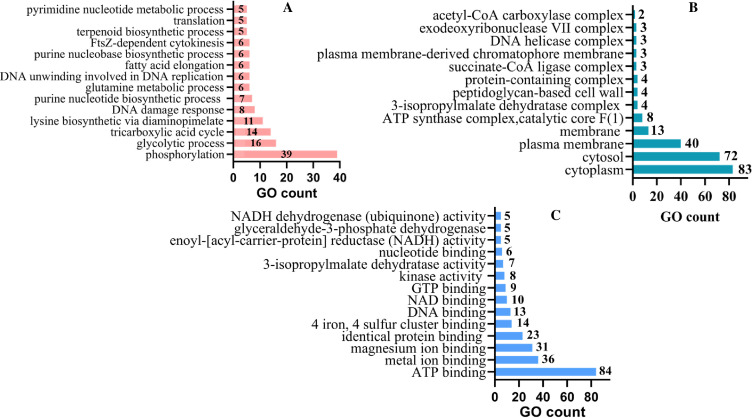
The comprehensive analysis of GO term frequencies within the UniProtKBs data set. This analysis provides valuable insights into the prevalence and distribution of GO terms across three categories: (**A**) BP, (**B**) CC, and (**C**) MF.

The distribution of gene ontology terms across biological processes, cellular components, and molecular functions is depicted in Fig. 3A through C, respectively. In the BP category, a total of 146 unique GO terms were observed, and those with frequencies exceeding 5 are highlighted in the plot. For the CC category, a total of 47 unique GO terms were identified, and the plot focuses on CC terms with frequencies greater than 1. In the MF category, a total of 98 unique GO terms were discovered, with the plot highlighting MF terms that occur more frequently than 5. The highest number of enriched genes (#39) in BP belongs to phosphorylation process, whereas in CC, cytoplasmic and periplasmic genes were observed to be 83 and 40, respectively. ATP binding and metal ion binding carried a significant number of genes (#84 and #36, respectively). Therefore, the analysis of GO terms related to BP, MF, and CC yielded insightful results that highlighted the intricate interconnections between these domains. The visualization in Fig. S2 underscores the harmonious coordination and cooperation among the GO terms (from 256 core gene families), forming a complex network that drives cellular activities. The interlinkage observed among the GO terms motivates the rationale behind categorizing the 256 exact core genes into 31 distinct categories, as presented in Table S3.

**Fig 4:**
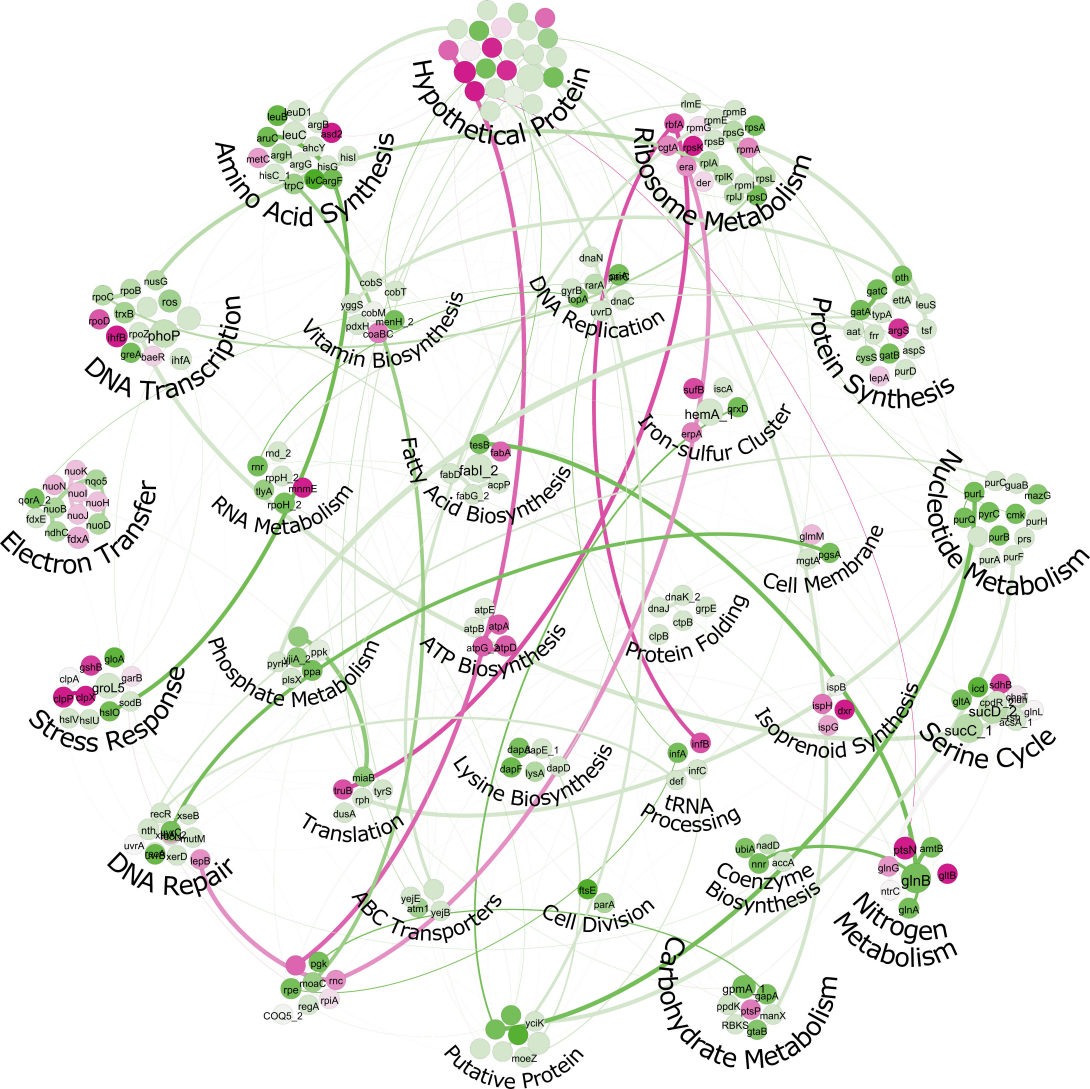
The interconnections among gene families visualized using Gephi. Colors indicate the degree of association between gene families based on modularity statistical scores. Green nodes represent the strongest associations, while pink nodes indicate weaker connections

The physical protein-protein interactions of the 256 core genes are represented in Fig. 4. To gain further insights into these interactions, a statistical analysis using Gephi was conducted, and the results are provided as Fig. S3. The analysis revealed some interesting findings regarding the connectivity of these proteins. Specifically, the average degree of connectivity among the proteins was calculated to be 1.6, indicating that, on average, each protein interacts with approximately 1.6 other proteins in the network. Additionally, the average weighted degree, which takes into account the strength of these interactions, was found to be 31.1.

A significant portion of conserved gene families identified in this study is associated with fundamental cellular processes, including DNA replication, transcription, and translation. These conserved gene families are regarded as indispensable components across various environmental conditions. Notably, we observed conservation of genes related to nitrogen metabolism, which aligns with previous reports highlighting the nitrogen-fixing capabilities of type II methylotrophs (14). Additionally, the presence of conserved genes involved in isoprenoid synthesis suggests potential roles in secondary metabolite production. However, it is important to note that conservation of these genes does not necessarily imply the complete functionality of the corresponding metabolic pathways across all 75 organisms. Intriguingly, our analysis revealed the presence of 22 conserved hypothetical gene families across all the 75 organisms, the putative functions of which are discussed in detail in the subsequent section.

### Functional annotation of exact core hypothetical genes

The functional annotation of exact core hypothetical genes represents a crucial step in unraveling the functional potential and understanding the significance of these genes within the broader context of the studied organisms or biological systems. Out of the 256 gene families in the exact core, a subset of 15 gene families remained unassigned in terms of their function. Table 1 presents the annotation results of hypothetical proteins, which might contribute valuable addition to the existing gene repertoire of methylotrophs. Table S4 provides a comprehensive overview of the detailed FASTA sequences and corresponding scores associated with the annotation of the hypothetical proteins.

**TABLE 1:** Annotation of hypothetical proteins

Gene family^*a*^	Annotation	GO function	Confidence^*b*^
*Methylobacterium_jeotgali*_CDS_4118	*rpmE*, 50S ribosomal protein L31	Translation	Yes
*Methylooceanibacter_caenitepidi*_CDS_2654	yhbS, N-acetyltransferase (GNAT)	Organonitrogen compound biosynthetic process	No
*Methylobacterium_phyllostachyos*_CDS_4295	NADH dehydrogenase (ubiquinone) 1 alpha subcomplex	Organophosphate metabolic process	Yes
*Methylocella_tundrae*_CDS_3415	EC:3.4.11.9: Xaa-Pro aminopeptidase	Metalloaminopeptidase activity	Yes
*Methylocystis_hirsuta*_CDS_2285	MoxR-like ATPase	Organonitrogen compound catabolic process/metalloendopeptidase activity	No
*Methylooceanibacter_superfactus*_CDS_2697	ATPase family	Cell cycle process	No
*Methylovirgula*_sp._HY1_CDS_2003	Glutamine amidotransferase	Pyrophosphatase activity	Yes
*Methylobacterium_adhaesivum*_CDS_1753	NA^*c*^	Cytoplasm	No
*Methylovirgula*_sp._4M-Z18_CDS_4185	RNA-binding domains	Lyase activity	No
*Methylobacterium_terrae*_CDS_3436	NA	Phosphatidylinositol phosphorylation	No
*Methylosinus_trichosporium*_OB3b_CDS_4175	Conserved protein YbaR	Enzyme inhibitor activity	No
*Methylocystis_hirsuta*_CDS_2870	Lysine decarboxylase	Quinone metabolic process	Yes
*Methylopila*_sp._Yamaguchi_CDS_1376	DNA polymerase III subunit	Binding	Yes
*Methylooceanibacter*_*marginalis*_CDS_2229	Type III secretion system	Nitrogen compound metabolic process	Yes
*Methylocystis_parvus*_CDS_0309	Polyhydroxyalkanoate synthesis repressor PhaR	rRNA processing/transcription factor binding	Yes
*Methylooceanibacter_methanicus*_CDS_0298	Polyhydroxyalkanoate synthesis regulator protein	rRNA processing/transcription factor binding	Yes
*Methylobacterium_brachythecii*_CDS_2235	Zn-finger domain	Cofactor binding	No
*Methylobacterium_bullatum*_CDS_2120	ATPase, AAA+	Transferase activity, transferring phosphorus-containing groups	No
*Methylobrevis_albus*_CDS_1240	Haloacid dehalogenase	Catalytic activity	Yes
*Methylocystis_parvus*_CDS_1055	SH3-like domain	Organonitrogen compound metabolic process	No
*Methylobacterium_oxalidis*_CDS_2397	PtsH, phosphotransferase	Carbohydrate transport	Yes
*Methylocapsa*_sp._S129_CDS_5161	Dipeptidyl aminopeptidase	Catalytic activity	Yes

^*a*^ Information related to the gene families with regard to the FASTA sequences used in the study are provided in Table S4.

^*b*^ The confidence level is determined by the combined score derived from motif-based protein-level prediction from PROSITE and NCBI, as well as the GO process-based ProteInfer.

^*c*^ NA: not applicable.

Our efforts to unravel the functionality of the type II methylotrophs led to the discovery of 12 gene families. Through our investigations, we were able to shed light on the functions of these 12 predicted proteins. 50S ribosomal protein L31 is involved in the assembly and stabilization of the ribosomal subunits, facilitating the translation of mRNA into functional proteins (36). The NADH dehydrogenase (ubiquinone) 1 alpha subcomplex, on the other hand, is part of the respiratory chain complex I, which is involved in energy production through oxidative phosphorylation (37). This subcomplex is responsible for the transfer of electrons from NADH to ubiquinone, a critical step in the electron transport chain. The Xaa-Pro aminopeptidase (EC:3.4.11.9) is involved in the cleavage of Xaa-Pro peptide bonds, contributing to protein degradation and turnover (38). Glutamine amidotransferase plays a key role in nitrogen metabolism by catalyzing the transfer of an amino group from glutamine to various acceptor molecules. Lysine decarboxylase participates in the decarboxylation of lysine, a process important for the synthesis of certain polyamines. The DNA polymerase III subunit is an essential component of the DNA replication machinery, contributing to accurate and efficient DNA synthesis (39). The type III secretion system enables the delivery of effector proteins from bacteria to host cells, playing a role in pathogenicity (40, 41). The polyhydroxyalkanoate (PHA) synthesis repressor PhaR regulates the synthesis of polyhydroxyalkanoates, which are storage compounds produced by bacteria. Additionally, the polyhydroxyalkanoate synthesis regulator protein controls the expression of genes involved in polyhydroxyalkanoate synthesis (42, 43). The haloacid dehalogenase enzyme is involved in the detoxification and degradation of halogenated organic compounds (44, 45). PtsH, also known as phosphotransferase, is a component of the sugar transport system, facilitating the phosphorylation of sugars for their uptake (46–48). Lastly, the dipeptidyl aminopeptidase enzyme participates in the cleavage of dipeptides, contributing to peptide metabolism.

### Structure of shell and cloud

The observation of 3,433 gene families in the shell fraction and a staggering 69,732 in the cloud fraction highlights substantial evidence for horizontal gene transfer (HGT) occurring across the taxonomy, emphasizing the potential influence of genetic exchange in shaping the evolutionary dynamics of these methylotrophic organisms. Figure S4 presents the distribution of these genes across various taxonomic groups and cellular metabolic pathways. This section serves as a brief overview of the observed trends, acknowledging the presence of genes from other taxonomic lineages in the shell and cloud regions.

Type II methylotrophs, which belong to the α-proteobacteria, exhibit an interesting trend in their taxonomic distribution. As we progress from the persistent core genes (Fig. S4A) to the shell genes (Fig. S4B) and finally to the cloud genes (Fig. S4C), we observe a gradual decrease in the proportion of α-proteobacteria. This observation can be attributed to the presence of genes from other taxonomic lineages in the shell and cloud regions. It is likely that type II methylotrophs have acquired these genes through horizontal gene transfer from other bacteria, allowing them to adapt and thrive in diverse environments by harnessing the genetic repertoire of different organisms. Furthermore, an interesting observation in our analysis is the increasing fraction of genes associated with environmental signaling processes from persistent to cloud, accompanied by a decrease in the fraction of genes related to cellular metabolism. This trend suggests that cellular metabolism genes are highly conserved within the core genomes of type II methylotrophs, while the genes originating from other species are found in the cloud and shell regions, exhibiting greater variability and lack of conservation within the type II methanotrophic community.

### Functional gene repertoire

#### Nitrogen-fixation capability

We have detected both molybdenum-iron (Mo-Fe) and vanadium-iron (V-Fe) nitrogenases across the diverse methylotrophic taxa investigated, spanning both terrestrial and atmospheric environments. Specifically, Mo-Fe nitrogenases were prevalent in 17 out of the 75 studied methylotrophic genera, including *Methylocella* spp., *Methylocystis* spp., *Methyloferula* spp., *Methylosinus* spp., *Methylovirgula* spp., and *Methylocapsa* spp. Concurrently, we identified the presence of the FixK transcriptional regulator, crucial for N_2_ fixation, in 17 organisms, encompassing *Methylocapsa* spp., *Methyloferula* spp., *Methylopila* spp., and *Methylobacterium* spp. Furthermore, among taxa possessing Mo-Fe nitrogenases, *Methylocella* spp., *Methylosinus* spp., and *Methylovirgula* spp. were observed to lack the *FixK* gene. However, *Methylocapsa* spp., *Methylocystis bryophila*, *Methylocystis heyeri*, and *Methylocystis parvus* were noteworthy for containing both Mo-Fe and V-Fe nitrogenases, with the presence of the *VnfA* transcriptional activator, indicative of specialized regulatory mechanisms in these organisms. Interestingly, our findings indicated that organisms harboring V-Fe nitrogenases lack the *FixK* gene.

#### Growth on C1 compounds

**Fig 5:**
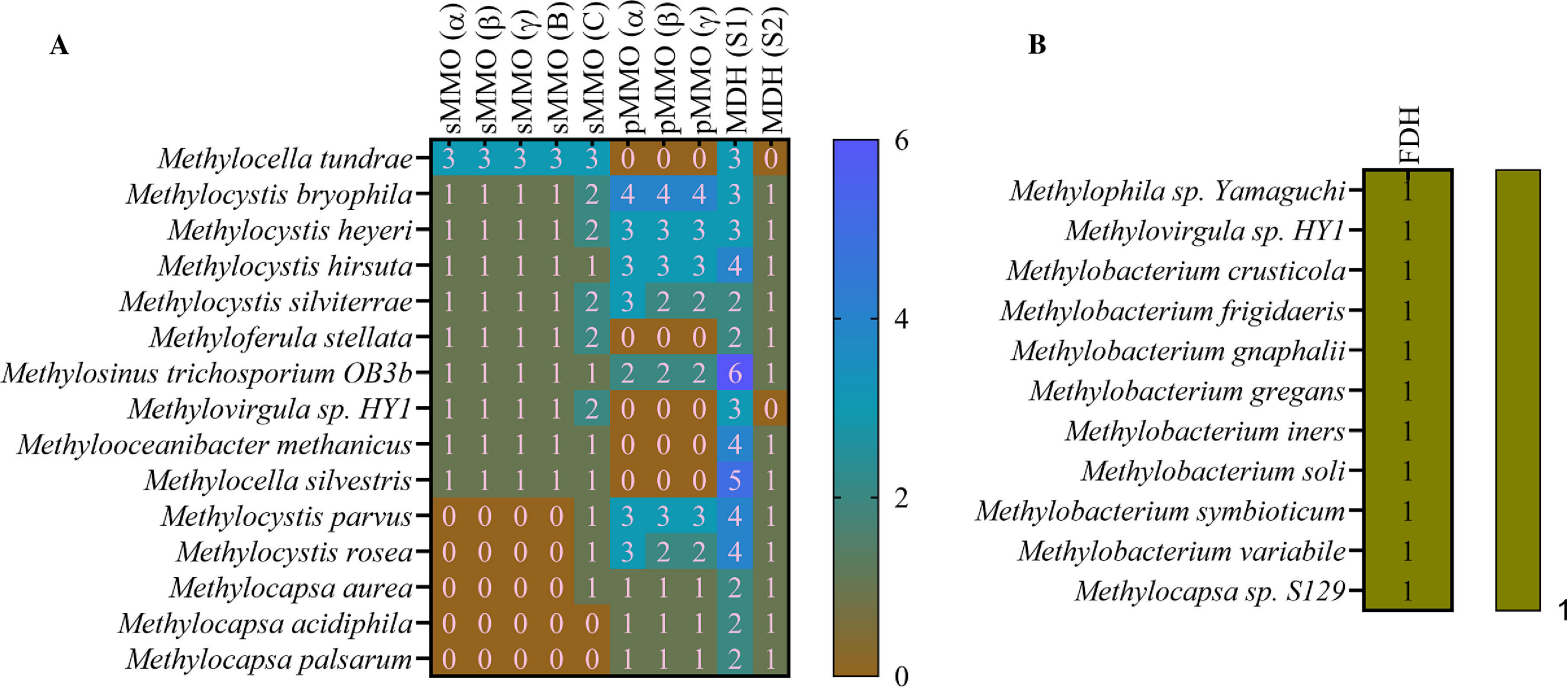
Presence-absence matrix (heatmap) illustrating the distribution of key genes involved in methane metabolism among the examined methanotrophic strains. Subfigure (**A**) displays the availability of genes associated with MMOs and MDH. Subfigure (**B**) focuses specifically on the presence or absence of formaldehyde dehydrogenase (FDH) genes.

In our study, we investigated the presence of specific enzymes involved in methane oxidation, namely sMMO and pMMO, in all the 75 organisms. The 15 out of 75 organisms having MMOs are shown in Fig. 5A. Our findings revealed interesting patterns regarding the distribution of these enzymes across different organisms. Among the organisms examined, we identified 10 organisms that contain the sMMO hydrolase (α, β, and γ chains), while 10 organisms contain sMMO protein B (regulatory). Additionally, 13 organisms were found to possess sMMO protein C (reductase). Notably, the three additional organisms identified as *Methylocystis parvus*, *Methylocystis rosea*, and *Methylocapsa aurea* specifically harbored sMMO protein C. It is worth mentioning that in contrast to a previous study that reported two copies, our findings revealed that *Methylocella tundrae* exhibits three copies of all genes associated with sMMO, which are non-identical to each other. On the other hand, *Methylocystis bryophila*, *Methylocystis hirsuta*, *Methylocystis silviterrae*, and *Methylosinus trichosporium* contained two copies of protein C. Moreover, *Methylocella tundrae*, *Methyloferula stellata*, *Methylovirgula* sp. HY1, and *Methylocella silvestris* lacked pMMO proteins but possessed sMMO proteins. However, we noted that *Methylocystis parvus*, *Methylocystis rosea*, and *Methylocapsa aurea*, in addition to protein C, also contained all the pMMO genes. Interestingly, *Methylocapsa aurea*, *Methylocapsa acidiphila*, and *Methylocapsa palsarum* exclusively harbored pMMO genes, with one copy each. Notably, *Methylocystis bryophila* displayed four copies of pMMO genes.

Furthermore, we observed the presence of two subunits of methanol dehydrogenase (cytochrome c) in the organisms examined. Subunit 1 was detected in 73 organisms, with an average copy number of 3.16 per organisms. On the other hand, subunit II was found in 55 organisms, with an average copy number of 1.05 per organism. It is noteworthy that *Methylovirgula* sp. 4M-Z18 and *Methylocapsa* sp. S129 did not exhibit any methanol dehydrogenase (cytochrome c). However, it is interesting to highlight that *Methylovirgula* sp. 4M-Z18 possesses NAD-dependent methanol dehydrogenase, indicating an alternative pathway for methanol oxidation in this particular organism. Figure 5A illustrates the distribution of methanol dehydrogenase subunits among the organisms that possess either pMMO or sMMO. This subset of organisms was selected to highlight the presence of methanol dehydrogenase, which is a key enzyme involved in methanol utilization.

The presence of formaldehyde dehydrogenase (FDH), which facilitates the direct oxidation of formaldehyde to formic acid, was observed in only 10 organisms (Fig. 5B). Interestingly, none of the examined organisms possessed either of sMMO or pMMO genes, except for *Methylovirgula* sp. HY1. This suggests that in methanotrophs, the direct oxidation of formaldehyde to formic acid may not be the primary pathway, and alternative assimilation pathways are likely in place, via the serine pathway. However, these 10 organisms that do possess FDH have the ability to utilize formaldehyde as a carbon source and can efficiently convert it to carbon dioxide. The metabolic flux in these organisms is directed toward carbon dioxide production, indicating the importance of formaldehyde metabolism in their carbon assimilation strategies. It is worth noting that among the 11 organisms, *Methylocapsa* sp. S129 stands out as it contains a non-identical form of formaldehyde dehydrogenase compared to the other nine organisms.

In summary, our study identified a total of 15 organisms (out of 75 organisms) capable of methane oxidation, classifying them as methanotrophs. Among these organisms, five contained only sMMO, five contained only pMMO, and five contained both types of enzymes. Notably, the *Methylocapsa* genus exclusively possessed pMMO, while *Methylocella* and *Methyloferula* solely exhibited sMMO. The distribution of sMMO copies among the 10 organisms was found to be 12, whereas the number of pMMO copies totaled 22. This observation suggests that organisms possessing pMMO may exhibit higher methane oxidation rates compared to those with sMMO. The detection of methanol dehydrogenase subunit 1 in the examined organisms suggests their ability to utilize methanol as a carbon source, except for *Methylocapsa* sp. S129. This finding implies that these organisms have the enzymatic machinery necessary for methanol oxidation, a characteristic trait of methanotrophs. However, it is intriguing to note that *Methylovirgula* sp. 4M-Z18 possesses NAD-dependent methanol dehydrogenase, indicating an alternative pathway for methanol oxidation. The exact nature and mechanism of methanol oxidation in *Methylovirgula* sp. 4M-Z18 warrant further investigation to elucidate the specific enzymatic reactions involved in this process.

#### Serine pathway

In our study, we made an intriguing observation regarding the presence of the key enzyme serine hydroxymethyltransferase (SHMT) in the examined organisms. We found that SHMT was present in 74 organisms, with an average copy number of 1.31 per organism, highlighting its prevalence and importance in methylotrophic metabolism. However, it is worth noting that *Methylooceanibacter marginalis* was an exception to this pattern, as it did not possess SHMT. An interesting finding emerged when examining *Methylobacterium variabile*, as it exhibited both isozymes of SHMT. This unique characteristic suggests that *Methylobacterium variabile* has the potential to utilize a broader range of carbon sources, including succinate, methane, and methanol. In our study, we made an intriguing discovery concerning the presence of serine-glyoxylate transaminase (SGT) in all 75 examined organisms. As far as our current knowledge extends, the literature does not thoroughly explore the isozymes of SGT. Nevertheless, in the course of our inquiry, we observed three distinct isozymes of this enzyme across various organisms. Of notable interest is the revelation concerning *Methylobrevis* sp., which harbors a distinctive SGT isozyme absent in all other studied organisms. Another notable observation is that *Methylobrevis albus* shares a common isozyme with 70 other organisms, indicating a conserved functional role for this particular isozyme among a significant subset of the organisms. Additionally, there are 11 more organisms that share the most common isozyme but also possess a non-identical isozyme, suggesting additional metabolic diversity within this group.

Our investigation revealed the ubiquitous presence of hydroxypyruvate reductase (HPR) in all 75 examined organisms but with 22 different isoforms. Several organisms, including *Methylocapsa* sp. S129, *Methylopila* sp. Yamaguchi, *Methylobacterium jeotgali*, and *Methylobacterium crusticola*, exhibited a distinct and entirely different type of the HPR enzyme, indicating the presence of unique functional adaptations in their glyoxylate and hydroxypyruvate metabolic pathways. Furthermore, a putative hydroxypyruvate reductase was observed in 73 organisms, with an average copy number of 1.14 per organism. Notably, *Methylobacterium ajmalii* displayed the highest number of copies of this enzyme, indicating a potentially significant role in the metabolic activities of this particular organism. Further investigations on the enzymes, glycerate 2-kinase, phosphoglycerate mutase, phosphopyruvate hydratase, phosphoenolpyruvate carboxylase, malate dehydrogenase, and malyl-CoA lyase, showed that these enzymes are indeed present in all the 75 organisms but were observed in the shell and cloud region due to their variations in isoforms.

#### Glyoxylate cycle and ethylmalonyl-CoA pathway

During our analysis of the isocitrate lyase enzyme, we made interesting observations regarding its presence among the methane-oxidizing organisms. Out of the 15 organisms capable of oxidizing methane, only 6 organisms, namely *Methyloferula stellata*, *Methylovirgula* sp. HY1, *Methylocapsa silvestris*, *Methylocapsa aurea*, and *Methylocapsa palsarum*, were found to possess the isocitrate lyase enzyme. This indicates that these organisms utilize the glyoxylate pathway for carbon assimilation. On the other hand, the remaining nine organisms may follow ethylmalonyl-CoA (EMC) pathway instead. Interestingly, among the methylotrophic organisms, *Methylobacterium gossipiicola*, *Methylovirgula ligni*, *Methylovirgula* sp. 4MZ18, and *Methylocapsa acidiphila* were found to possess isocitrate lyase, indicating their potential utilization of the glyoxylate pathway for carbon assimilation. Another key enzyme in the glyoxylate pathway is malate synthase. We further investigated the presence of malate synthase among these organisms. We observed that malate synthase was present in 31 organisms, either in the G-form or the A-form. Notably, all the species mentioned above that possess isocitrate lyase were also found to contain malate synthase, except for *Methylobacterium gossipiicola*, suggesting the presence of the glyoxylate pathway in the six methanotrophic strains. Among these organisms, *Methylocapsa* sp. S129 was the only one to possess the A-form of malate synthase, while the rest had the G-form. Interestingly, *Methylovirgula* sp. HY1 and *Methyloferula stellata* not only possessed the common G-form of malate synthase but also exhibited distinct G-forms in their genomes.

Based on our expectation that nine organisms possess the glyoxylate pathway, we hypothesized that the remaining 66 organisms would likely follow the ethylmalonyl-CoA pathway. To explore this further, we focused on the central coenzyme, crotonyl-CoA carboxylase/reductase. Interestingly, our analysis revealed the presence of crotonyl-CoA carboxylase/reductase in 67 organisms, with an average copy number of 1.03. This finding aligned with our expectation that the majority of the organisms would utilize the EMC pathway. It was intriguing to note that the methylotrophic strain *Methylovirgula* sp. 4MZ18 possessed both the enzymes associated with the glyoxylate pathway and the EMC pathway. Another enzyme of interest within the EMC pathway is methylsuccinyl-CoA dehydrogenase. We observed the presence of this enzyme in all 67 organisms, consistent with the presence of crotonyl-CoA carboxylase/reductase. However, among these organisms, 33 exhibited a distinct isozyme copy of this enzyme. Notably, *Methylobacterium gossipiicola* was found to contain both crotonyl-CoA carboxylase/reductase and methylsuccinyl-CoA dehydrogenase, indicating its adherence to the EMC pathway, despite the presence of isocitrate lyase within its genome.

### Phylogenetic analysis

**Fig 6:**
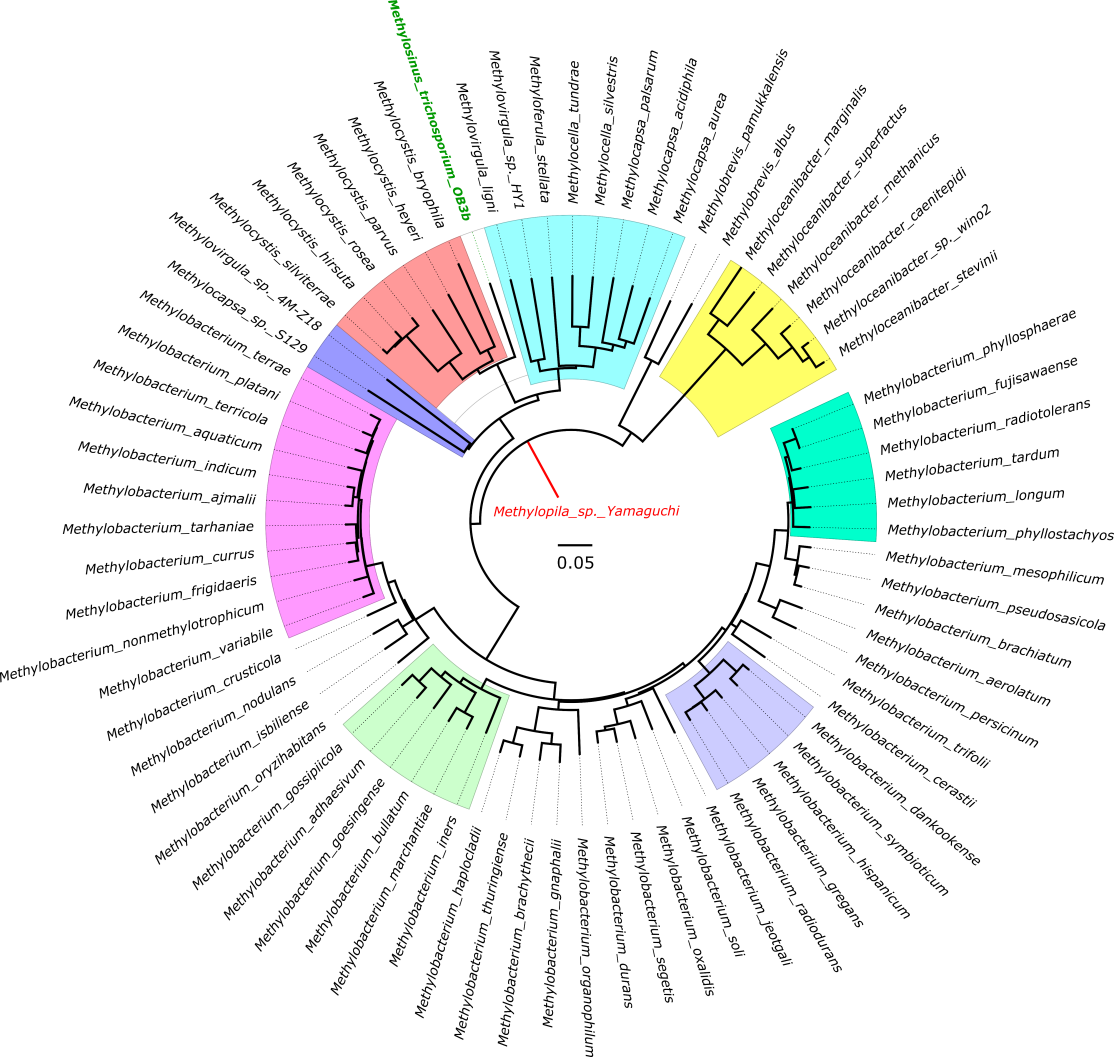
Dendrogram illustrating the hierarchical relationships among 75 type II methylotrophs. Branch lengths scaled at a ratio of 1 cm = 0.05, ensuring precise clustering of evolutionarily related methylotrophs. The outgroup is highlighted in red letters, while the methanotroph of interest is denoted by green letters. Noteworthy clads are highlighted clusters of closely related methylotrophs.

The phylogenetic tree analysis revealed intriguing patterns of classification among the organisms. Two major divisions were observed (shown in Fig. 6), with one comprising the *Methylobacterium* genus and the other consisting of the remaining genera. The genetic patterns observed in relation to the enzymes shed light on the unique characteristics of *Methylobrevis* compared to other methylotrophs during formaldehyde assimilation. *Methylobrevis* exhibited distinct isozymes or isoforms of enzymes present in other organisms, setting it apart from the common methanotrophic clade. Notably, *Methylocapsa* sp. S129 displayed a distinct genomic architecture and was separated from other *Methylocapsa* species. This separation was evident through the absence of methanol dehydrogenase in this particular methylotroph. Despite this distinction, *Methylocapsa* sp. S129 showed high similarity with *Methylovirgula* sp. 4M-Z18. It is worth mentioning that *Methylovirgula* sp. 4M-Z18 was also separated from other *Methylovirgula* species. Given the interconnectivity and lack of association with other organisms, we propose the establishment of a new separate genus for *Methylocapsa* sp. S129 and *Methylovirgula* sp. 4M-Z18.

A cluster comprising the *Methylocapsa*, *Methyloferula*, *Methylocella*, and *Methylovirgula* genera was observed (highlighted in bluish green). Additionally, the model organism *Methylosinus trichosporium* OB3b formed a common cluster (highlighted in orange) with the *Methylocystis* genus. It is evident that the organisms within the bluish green cluster and the orange cluster are primarily methanotrophs, with the exception of *Methylovirgula ligni*. The *Methylooceanibacter* genus displayed a distinct cluster without association to other organisms, indicating that all species within this genus are closely related. However, an exception occurred with *Methylooceanibacter methanicus*, which exhibited the ability to oxidize methane. A cluster highlighted in pink contained *Methylobacterium* species with no significant distance between the genera. It is worth noting that *Methylobacterium ajmalii*, despite being reported for its methane-oxidizing capacity, did not have any mapped genes within the pangenome analysis. This suggests the need for a comprehensive search within the genome, as a distinct isoform of MMOs may be present. Similar considerations apply to its sister clade, *Methylobacterium indicum*.

While our focus primarily revolved around major pathways such as methane oxidation, the serine pathway, glyoxylate pathway, and EMC pathway, it is essential to give due attention to other pathway genes. Understanding the capabilities of these type II methylotrophs to grow on various C1-C6 compounds requires a comprehensive investigation of additional pathways. The observed variations among these organisms highlight the diversity and adaptability within this group of microorganisms, emphasizing the need for further exploration and characterization of their unique metabolic capabilities.

## DISCUSSION

### Exact core genes

This investigation underscores the significance of conserved gene families in driving essential cellular processes such as DNA replication, transcription, and translation, which are indispensable for cellular survival across diverse environmental conditions. These processes are tightly interconnected, as evidenced by the strong interconnections revealed by GO terms, in terms of BP, MF, and CC. For instance, phosphorylation, a critical post-translational modification, is intricately linked to key metabolic pathways like glycolysis and the tricarboxylic acid cycle (TCA), pivotal for energy production (49, 50). Chistoserdova et al. documented the occurrence of complete TCA cycles, along with a full serine cycle, in a very few type II methylotrophs, notably including *Methylobacterium extorquens* and *Granulibacter bethesdensis* (51). However, in addition to this, *M. trichosporium* OB3b possesses a closed and reversible TCA cycle capable of channeling TCA intermediates toward pyruvate, acetyl CoA, and manoyl-CoA (52). Nonetheless, the interconnectedness of the EMC pathway, serine cycle, and TCA cycle confers an advantageous control mechanism over carbon flux within the TCA cycle (53). Similarly, the DNA damage response pathway, crucial for maintaining genomic integrity, intertwines with DNA replication and topological changes (54, 55). Additionally, biosynthetic processes such as nucleotide biosynthesis, including purine and pyrimidine pathways, are interconnected, playing pivotal roles in DNA and RNA synthesis (56, 57). Furthermore, the investigation highlights the interplay between various biosynthetic pathways, such as lysine biosynthesis and fatty acid elongation, elucidating the intricate relationship between amino acid metabolism and lipid synthesis (58–60). Processes like FtsZ-dependent cytokinesis underscore the importance of cellular division, while terpenoid biosynthesis demonstrates the interconnectedness between metabolic processes and antioxidant defense mechanisms (61, 62).

Turning to molecular function counts, the analysis reveals crucial binding events, including ATP and GTP binding, which are central to cellular energy transfer processes (63). Metal ion binding, particularly magnesium ion binding, underscores the importance of metal ions as cofactors in enzymatic reactions and for maintaining protein structural integrity (64–66). Moreover, protein-protein interactions, exemplified by identical protein binding and 4 iron-4 sulfur cluster binding, are vital for protein complex formation and electron transfer processes (67–69). Enzymatic activities like NAD binding and glyceraldehyde-3-phosphate dehydrogenase activity are pivotal for redox reactions and glycolysis, respectively, highlighting the diverse roles of proteins in cellular function and metabolism (70). Enzymes, such as NADH dehydrogenase (ubiquinone) activity and enoyl-[acyl-carrier-protein] reductase (NADH) activity, rely on NADH as a cofactor to catalyze specific reactions (71).

In addition, the study uncovered 22 hypothetical genes that were conserved among all 75 methylotrophs examined. Notably, during the annotation process, 2 enzymes stood out prominently: PhaR and haloacid dehalogenase, within the subset of 12 gene families. The Akira et al. study demonstrated that the presence of PhaR in an organism correlates with the production of short-chain polyhydroxyalkanoates (43). In our investigation, the presence of PhaR indicates that type II methylotrophs possess the capability to produce short-chain PHAs. Again, methylotrophs are recognized for their effectiveness in bioremediating halogenated compounds, underscoring the significance of the presence of haloacid dehalogenase (72).

### Functional gene repertoire in shell and cloud

#### Nitrogen-fixation capability

Type II methylotrophs exhibit a remarkable trait of expressing nitrogenase (encoded by *nifH*) to harness atmospheric N_2_ as a nitrogen source, a pivotal adaptation in their ecological niche (14, 73). Auman et al. investigated this trait by assessing type II NifH amplification and acetylene reduction activity, confirming N_2_-fixing capability in *Methylocystis* spp. and *Methylosinus* spp. (14). Type II methanotrophs, exemplified by *Methylosinus* and *Methylocella*, are believed to play a significant role in N_2_ fixation within forest soil ecosystems and rice plants (74). Alongside them, diazotrophs like *Rhizobium*, *Bradyrhizobium*, and *Mesorhizobium* are renowned for their N_2_-fixing abilities and are commonly found in association with methylotrophs in rice paddies (75). While Mo-Fe nitrogenases are ubiquitous among diazotrophs, V-Fe nitrogenases are confined to a select group of prokaryotes, notably prevalent in *Methylocystis* strains isolated from wetlands (76). Notably, Mo-Fe nitrogenase is favored in Mo-rich conditions, whereas V-Fe nitrogenase predominates in Mo-deficient environments, with Oshkin et al. proposing this diversification as an adaptation to nutrient limitation in wetlands (15, 77). At standard temperatures (e.g., 30°C), V-Fe nitrogenase exhibits lower specific activity compared to its Mo-Fe counterpart, but at colder temperatures (e.g., 5°C), V-Fe nitrogenase surpasses Mo-Fe nitrogenase in activity (78). Moreover, V-Fe nitrogenase displays versatility by catalyzing both CO and N_2_ reductions, suggesting a potential interplay between carbon and nitrogen cycles in wetlands. Recent findings indicate the presence of V-Fe nitrogenase in *Methylocystis bryophila* S285 and *Methylospira mobilis* Shm1, although classical Mo-Fe nitrogenase genes are commonly found across methanotrophic genomes (79, 80).

We identified the presence of both V-Fe and Mo-Fe nitrogenases in *Methylocystis bryophila*, *Methylocystis heyeri*, and *Methylocystis parvus*, underscoring their significance in N_2_ fixation, which can be finely tuned simply by adjusting the growth medium. Furthermore, our investigation reveals the presence of the *fixK* gene, a transcription factor belonging to the Crp family, which governs N_2_ fixation, exhibiting both positive and negative regulation, mirroring findings in *Rhizobium meliloti*. In our study, we detected this gene in 15 methylotrophs, including prominent methanotroph strains*—Methylocapsa*, *Methylocella*, *Methyloferula*, and *Methylopila*. Of particular interest, strains associated with V-Fe nitrogenases—namely, *Methylocystis bryophila*, *Methylocystis heyeri*, and *Methylocystis parvus*—harbor the *VnfA* transcriptional activator as their N_2_-fixation regulatory protein, indicating a specialized adaptation within this subset of methanotrophs.

#### Growth on C1 compounds

The carbon assimilation pathways of aerobic methylotrophs that are capable of growing on C1 compounds as their source of carbon exhibit distinct mechanisms with regard to its enzyme characteristics (presence/absence of genes) (81). Methylotrophs typically rely on more commonly available C1 compounds such as methane, methanol, formaldehyde, or formic acid (82). Methanotrophs, a subset of methylotrophs, utilize methane as their primary substrate, which is oxidized by MMOs (sMMO or pMMO) to produce methanol (81). Among the type II methanotroph strains, *Methylocapsa aurea* exclusively possesses the pMMO form, while some strains exhibit either both forms of MMO (*Methylosinus trichosporium* OB3b) or solely sMMO (*Methylocella* and *Methyloferula*) (83, 84). In the case of *Methylocella tundrae*, it contains two similar but not identical sMMO operons, showcasing a 96% nucleotide identity between the mmoXYBZDCRG operons and an 88%–99% amino acid identity between the corresponding polypeptides (85). Although many methanotrophs harbor multiple copies of the *pmoCAB* operon, the existence of additional copies of the sMMO genes is quite not general (86). While, in our study, we identified three copies of the sMMO subunits (XYZ) within *Methylocella tundrae*, along with proteins B and C, deviating from the expected two copies.

The further oxidation of methanol in methylotrophs involves the action of methanol dehydrogenase, which converts methanol to formaldehyde (87). The calcium-dependent MDH is encoded by the *Mxa* operon, with its larger and smaller subunits denoted as mxaF and mxaI, respectively (88). This form is widespread among methanotrophs and is activated by the cytochrome c electron acceptor. In contrast, the lanthanide-dependent MDH, *xoxF*, dominates in acidophilic or acid-tolerant methylotrophs due to lanthanides’ stronger Lewis acid properties compared to calcium (89). This enhances the electrophilic nature of active carbons in PQQ, facilitating electron removal from methanol. In our study, among methanotrophs, *Methylovirgula* sp. 4M-Z18 exhibited NAD-dependent MDH, which donates electrons under both aerobic and anaerobic conditions, distinguishing it from cytochrome c-dependent MDH. Notably, this trait is commonly associated with gram-positive bacteria. The reports highlighted that *Methylosinus trichosporium* OB3b contains both xoxF and mxaF, a finding corroborated in our study (90). Additionally, we observed *Methylocella* spp. and *Methyloferula* spp. under the same category, which have not been reported previously.

The subsequent assimilation of formaldehyde in type II methylotrophs primarily relies on the serine cycle. This cycle allows the incorporation of formaldehyde into cellular metabolism for the synthesis of essential biomolecules (91). To complete the oxidation process, formaldehyde is further oxidized to formic acid by the action of formaldehyde dehydrogenase (92). Hence, methylotrophs derive the energy necessary for their growth by oxidizing C1 substrates through specific dehydrogenases. Unlike other organisms, they do not depend on a complete Krebs TCA cycle for their energy generation.

#### Serine pathway

The serine cycle is different from the other pathways in having carboxylic and amino acids as intermediates instead of the usual carbohydrates. In the initial step of the serine pathway, formaldehyde undergoes a reaction with glycine to produce L-serine. This crucial transformation is facilitated by the enzyme glycine/serine hydroxymethyltransferase, which is classified as EC 2.1.2.1. SHMT utilizes tetrahydrofolate as a cofactor in this reaction. When formaldehyde binds to SHMT, it generates a complex called 5,10-methylenetetrahydrofolate. During the enzymatic process, the formaldehyde moiety from 5,10-methylenetetrahydrofolate is transferred to glycine, leading to the formation of L-serine. In a study conducted by O’Connor et al., it was demonstrated that facultative methylotrophs possess multiple isozymes of SHMT (93). Interestingly, the presence and activity of these isozymes were found to vary depending on the carbon source used for growth. Specifically, one isozyme was found to be predominant when the organism was grown using methane or methanol, while the other isozyme dominated when succinate was utilized as the sole carbon source. This study identified multiple isozymes of SHMT, directly linked to the organism’s capacity for multi-substrate uptake.

In the subsequent step of the pathway, L-serine undergoes transamination with glyoxylate, utilizing the enzyme serine-glyoxylate transaminase (EC 2.6.1.45). This reaction leads to the production of 3-hydroxypyruvate and glycine. The glycine generated can be recycled and serve as a substrate for SHMT. Meanwhile, hydroxypyruvate is reduced to D-glycerate by the enzyme hydroxypyruvate reductase (EC 1.1.1.81). The resulting D-glycerate is further phosphorylated by glycerate 2-kinase (EC 2.7.1.165) to yield 2-phospho-D-glycerate. At this stage, Samanta et al. showed the bifurcation of the pathway in *M. trichosporium* OB3b, which is consistent for a few other type II methylotrophs (especially *Methylocystis* sp.) (94–96). A portion of the 2-phospho-D-glycerate is converted by phosphoglycerate mutase (2,3-diphosphoglycerate dependent) (EC 5.4.2.11) into 3-phospho-D-glycerate. The remainder of the 2-phospho-D-glycerate is transformed by phosphopyruvate hydratase (EC 4.2.1.11) into phosphoenolpyruvate. Phosphoenolpyruvate carboxylase (EC 4.1.1.31) subsequently facilitates the fixation of carbon dioxide, converting phosphoenolpyruvate into oxaloacetate. Oxaloacetate is then reduced to (S)-malate by the enzyme malate dehydrogenase (EC 1.1.1.37). A reaction catalyzed by malate-CoA ligase (EC 6.2.1.9) forms malyl coenzyme A, which is further cleaved by malyl-CoA lyase (EC 4.1.3.24) into acetyl-CoA and glyoxylate (97). The presence of the last two enzymes (EC 6.2.1.9 and EC 4.1.3.24), as well as EC 1.1.1.81 and EC 2.7.1.165, in methylotrophs signifies the existence of the serine pathway.

#### Glyoxylate and EMC pathway

The fate of acetyl-CoA in an organism depends on the presence or absence of the enzyme isocitrate lyase (EC 4.1.3.1), which serves as a key enzyme in the glyoxylate cycle. Indeed, some methylotrophs do have the key enzyme (isocitrate lyase) of that pathway, and they assimilate C1 compounds by what is known as the icl+ serine cycle. If the organism possesses this enzyme, acetyl-CoA is converted to glyoxylate through the glyoxylate cycle, a modified version of TCA cycle (98). The glyoxylate cycle requires two key enzymes, namely isocitrate lyase and malate synthase, in addition to certain TCA cycle enzymes (99). These enzymes are often referred to as anaplerotic enzymes since they play a crucial role in replenishing the intermediates of the TCA cycle. As a result, this pathway is commonly known as the glyoxylate bypass (100). However, it had been shown previously that there is no isocitrate lyase present during methylotrophic growth for majority of methylotrophs. However, in the absence of isocitrate lyase, acetyl-CoA is processed through the EMC pathway (98).

Within EMC pathway, a C4 compound known as acetoacetyl-CoA, derived from two acetyl-CoA molecules, undergoes a series of transformations to yield a C5 compound called 2-methylfumaryl-CoA (101). This conversion involves the hydration of 2-methylfumaryl-CoA to produce (2R,3S)-β-methylmalyl-CoA. The latter compound is then cleaved into glyoxylate and propanoyl-CoA. By condensing glyoxylate with another molecule of acetyl-CoA, (S)-malate is formed (102). Simultaneously, propionyl-CoA undergoes carboxylation via a dedicated pathway, leading to the production of succinate (103). The central enzyme in this pathway is crotonyl-CoA carboxylase/reductase, which exhibits the remarkable ability to carboxylate and reduce the four-carbon compound crotonyl-CoA simultaneously (104). This enzymatic reaction results in the formation of a five-carbon compound known as (2S)-ethylmalonyl-CoA. In certain methylotrophs, an enzyme called ethylmalonyl-CoA mutase plays a pivotal role in the conversion of acetyl-CoA to glyoxylate (105). This unique enzyme belongs to a distinct category of coenzyme B12-dependent acyl-CoA mutases. Alongside, propionyl-CoA carboxylase is also involved in this process (106). Furthermore, the ethylmalonyl-CoA pathway for acetate assimilation is finalized by the action of methylsuccinyl-CoA dehydrogenase (107).

### Phylogenetic analysis

The genus *Methylobacterium* encompasses a diverse group of pink-pigmented facultatively methylotrophic bacteria (108). *Methylobacterium* demonstrate the ability to utilize one-carbon compounds such as formate, formaldehyde, and methanol as their sole carbon and energy source (109). Furthermore, they exhibit versatility in utilizing multi-carbon growth substrates. They have evolved to thrive in a wide range of environmental conditions, including extremes of temperature, salinity, drought, and pH, as well as acidic and alkaline habitats (110). These bacteria are commonly found in agroecosystems and can be isolated from various parts of plants (111). *Methylobacterium* play a crucial role in providing essential nutrients (nitrogen- and phosphorus-associated compounds) to plants during stressful periods, thereby enhancing their tolerance to abiotic stress (112). Multiple reports have shed light on the genetic and environmental diversity within the type II methylotrophs, revealing intriguing characteristics (113). One example is the *Methylobrevis* genus, which consists of two known species: *Methylobrevis albus* and *Methylobrevis pamukkalensis*. Of the two, *Methylobrevis pamukkalensis* stands out as a facultative halotolerant methylotroph (114). The classification of *Methylobacterium* within the type II methylotrophy and its role as a methanotroph have been the subject of extensive debate (115). The type species of *Methylobacterium*, e.g., *Methylobacterium organophilum,* was described for the first time as a facultative methane-utilizing bacterium (116). Notably, Bijlani et al. conducted a study demonstrating the growth of *Methylobacterium ajmalli* using both methane and methanol (117). Consequently, *Methylobacterium* species are often harnessed for metabolite production due to their capacity to grow on methane and its oxidation by-products, such as methanol and formaldehyde (118). However, the methanotrophy capacity of the remaining *Methylobacterium* species is yet to be fully elucidated. The genetic architecture of these bacteria explored through the phylogenetic analysis revealed a separate group from methanotrophs.

Hence, this study delves into the adaptability of methylotrophs—from their diverse substrate preferences to their distinct genetic architecture—for thriving in various environmental conditions and contributing to bioremediation efforts. In the future, a comprehensive analysis of individual isozymes involved in methane oxidation and the serine pathway across diverse methylotrophs will offer insights into variations in catalytic structure, enzyme-substrate conformations, and reaction mechanisms.

### Conclusion

Our pangenomic approach has provided valuable insights into the metabolic genetic distributions across the 75 type II methylotrophic strains. We identified 256 exact core gene families, encompassing essential housekeeping genes associated with central dogma processes, with the exception of 22 hypothetical proteins whose functions were elucidated. Notably, among the 12 identified hypothetical proteins, 1 was involved in translation, 1 related to nitrogen metabolism, and 2 with catalytic activity (haloacid dehalogenase and dipeptidyl aminopeptidase), showcasing their significance.

Our analysis of the shell and cloud regions revealed a trend of HGT, indicating the acquisition of a larger fraction of genes in the cloud region from other taxonomic communities. This highlights the adaptive strategy of type II methylotrophs to thrive in versatile environments by incorporating genetic material from diverse sources. Furthermore, our study unraveled a diverse repertoire of genes associated with crucial metabolic pathways, including methane oxidation, serine pathway, glyoxylate pathway, and ethylmalonyl-CoA pathway. We made intriguing observations, such as *Methylocella tundrae*’s possession of three copies of sMMO components, distinguishing it from other methanotrophs. Additionally, *Methylooceanibacter marginalis* lacked SHMT, while *Methylobacterium variabile* exhibited both isozymes of SHMT, suggesting their potential to utilize a broader range of carbon sources.

Phylogenetic analysis and distinct clustering patterns among the type II methylotrophs led us to propose a separate or possibly new genus for *Methylovirgula* sp. 4M-Z18 and *Methylocapsa* sp. S129, underscoring their unique characteristics and evolutionary divergence within the methylotrophic community. By unraveling the genetic foundations of type II methylotrophs, their potential for sustainable solutions in various fields, including bioremediation, biofuel production, and carbon capture, can be unlocked. These findings contribute to the advancement of environmental and industrial biotechnology, offering promising avenues for harnessing the metabolic capabilities of these organisms in practical applications.

## Data Availability

The original contributions presented in the study are included in the article and supplemental material. The PPanGGOLiN algorithm is available for access at https://github.com/labgem/PPanGGOLiN.

## SUPPLEMENTAL MATERIAL

Figure S1 msystems.00248-24-s0001.tiff Comprehensive visualization of the screening process, depicting the evaluation of each genome's completeness percentage.

Figure S2 msystems.00248-24-s0002.tiff Visualization of the intricate interconnections and collaborative relationships among the GO terms.

Figure S3 msystems.00248-24-s0003.tiff Statistical analysis of the 256 nodes determined using Gephi.

Figure S4 msystems.00248-24-s0004.tiff Distribution of genes across taxonomic groups and cellular metabolic pathways.

Legends msystems.00248-24-s0005.docx Legends to supplemental figures and tables.

Table S1 msystems.00248-24-s0006.xlsx The complete data set comprising 75 genomes, including information on genome size, GC content, genome coverage, and isolation site.

Table S2 msystems.00248-24-s0007.xlsx Detailed UniProt entries and descriptions of 256 exact core gene families.

Table S3 msystems.00248-24-s0008.xlsx Categorizing the 256 exact core genes into 31 distinct categories with their annotation, number of organisms, and number of sequences.

Table S4 msystems.00248-24-s0009.xlsx A comprehensive overview of the detailed FASTA sequence, and corresponding scores associated with the annotation of the hypothetical proteins.

ASM does not own the copyrights to Supplemental Material that may be linked to, or accessed through, an article. The authors have granted ASM a non-exclusive, world-wide license to publish the Supplemental Material files. Please contact the corresponding author directly for reuse.
