# Supplementary figures and images for: From genome to evolution: investigating type II methylotrophs using a pangenomic analysis
Source: mSystems. 2024 May 2;9(6):e00248-24. doi: 10.1128/msystems.00248-24 (PMC11237726; doi:10.1128/msystems.00248-24)

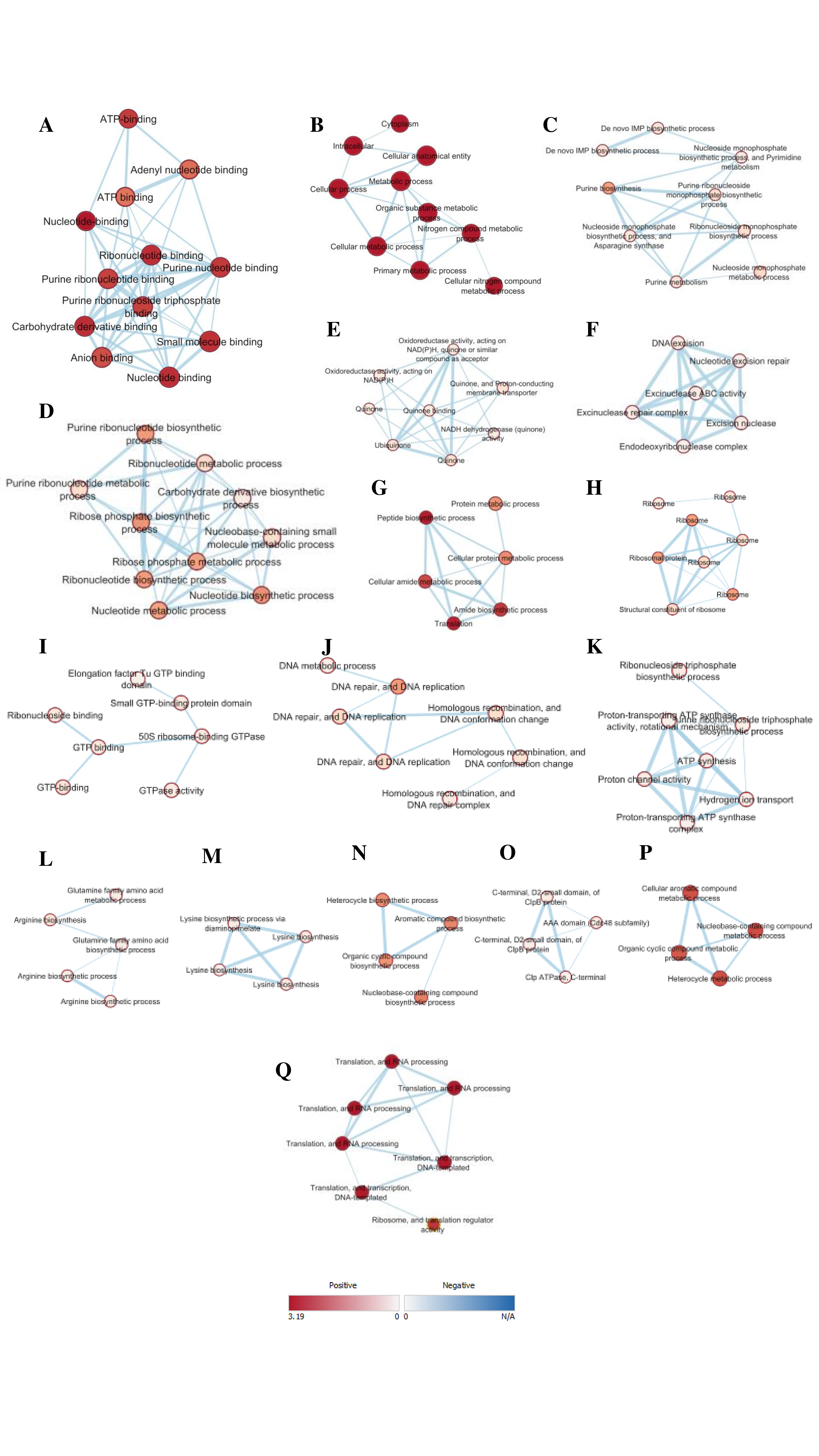

Supplement: Figure S2 — Visualization of the intricate interconnections and collaborative relationships among the GO terms. [file msystems.00248-24-s0002.tiff]

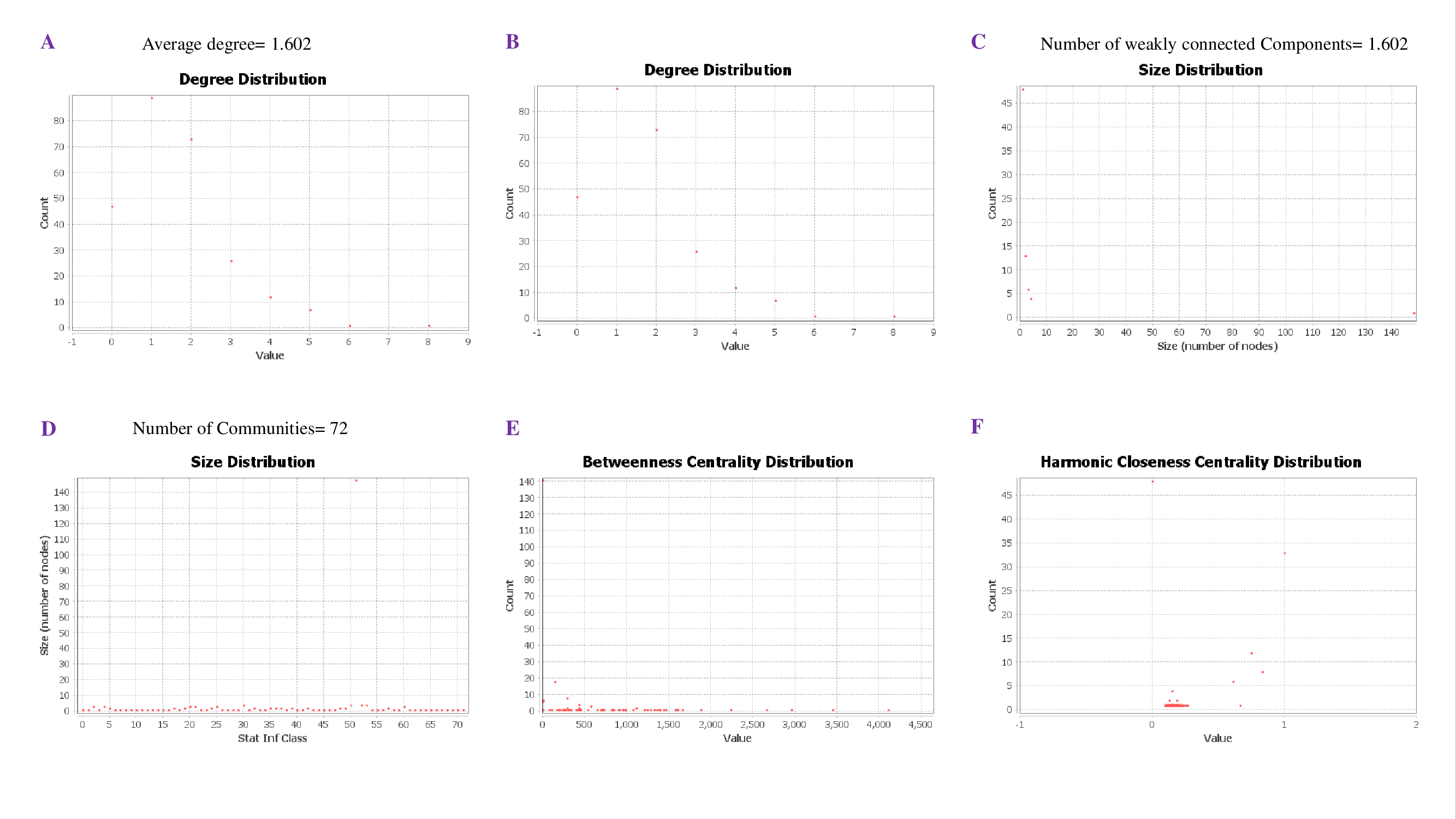

Supplement: Figure S3 — Statistical analysis of the 256 nodes determined using Gephi. [file msystems.00248-24-s0003.tiff]

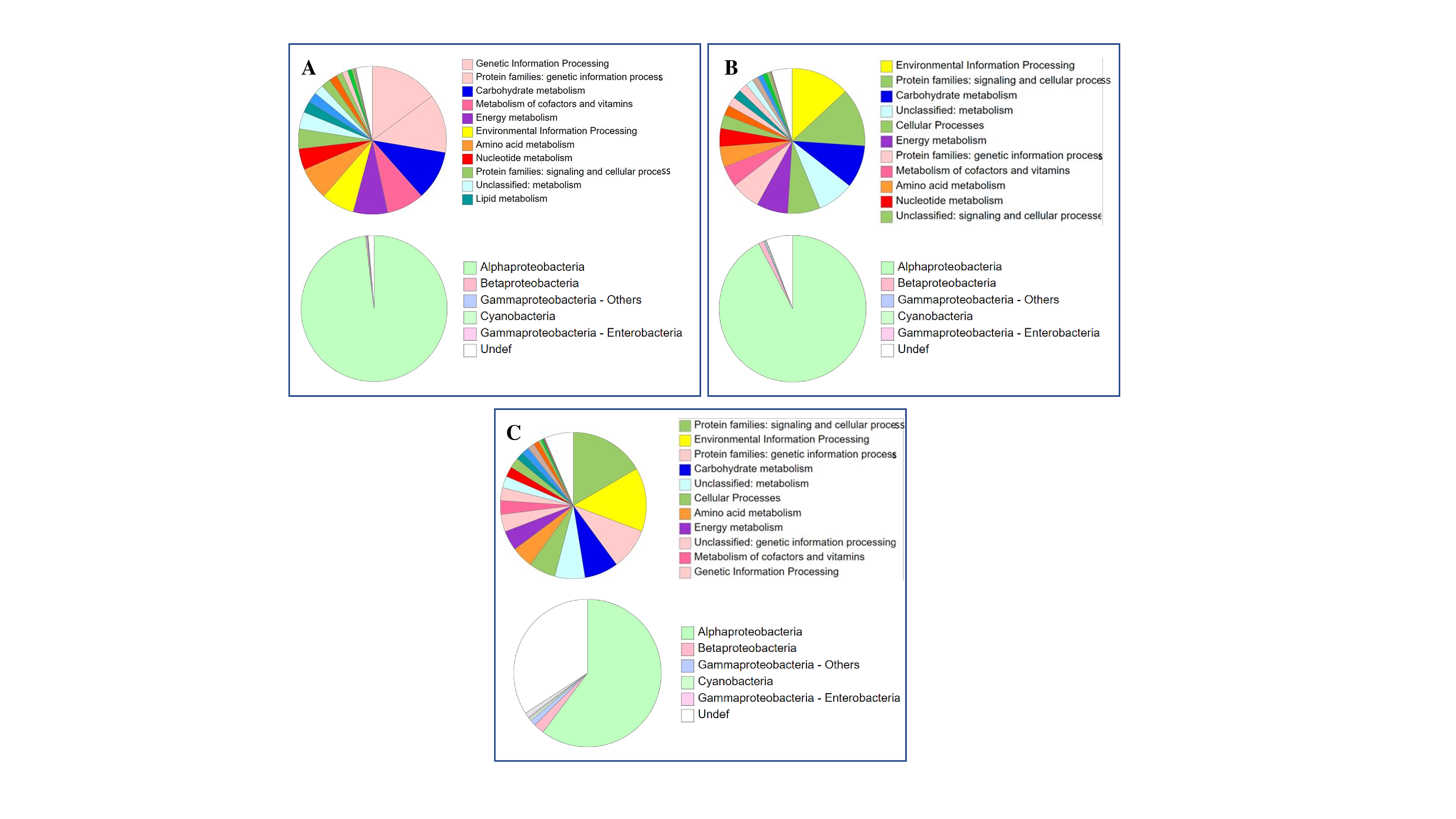

Supplement: Figure S4 — Distribution of genes across taxonomic groups and cellular metabolic pathways. [file msystems.00248-24-s0004.tiff]
