# Supplementary material for: From genome to evolution: investigating type II methylotrophs using a pangenomic analysis
Source: mSystems. 2024 May 2;9(6):e00248-24. doi: 10.1128/msystems.00248-24 (PMC11237726; doi:10.1128/msystems.00248-24)
Supplement: Legends — to supplemental figures and tables. [file msystems.00248-24-s0005.docx]

**Supplementary Material**

**From Genome to Evolution: Investigating Type II methylotrophs using a pangenomic analysis**

Dipayan Samanta^1,2^, Shailabh Rauniyar^1,3^, Priya Saxena^1,4^, and Rajesh K. Sani^1,2,3,4*^

**Figure S1:** Provides a comprehensive visualization of the screening process, depicting the evaluation of each genome's completeness percentage.

**Figure S2:** Visualization of the intricate interconnections and collaborative relationships among the GO terms (derived from 256 core gene families), revealing a complex network that orchestrates cellular activities (BP, MF, and CC). The node heatmap coloration highlights the strength of interactions, with vibrant red indicating strong associations and blue representing weaker connections. **(A)** ATP binding (MF), **(B)** cytoplasm (CC), **(C)** nucleoside biosynthesis (BP), **(D)** biosynthetic process (BP), **(E)** oxidoreductases (MF), **(F)** DNA excision repair (MF), **(G)** protein metabolism (BP), **(H)** ribosome subunits (BP), **(I)** GTP metabolism (MF), **(J)** DNA repair and replication (MF), **(K)** ATP synthesis (MF), **(L)** glutamine and arginine biosynthesis (BP), **(M)** lysine biosynthesis (BP), **(N)** organic biosynthesis (BP), **(O)** Atpase transporters (MF), **(P)** aromatic and heterocyclic biosynthesis (BP), **(Q)** translation (MF).

**Figure S3:** Statistical analysis of the 256 nodes determined using Gephi; **(A)** average degree, **(B)** average weighted degree, **(C)** connected components, **(D)** community size distribution, **(E)** betweeness centrality distribution, and **(F)** harmonic closeness centrality distribution.

**Figure S4:** Distribution of genes across taxonomic groups and cellular metabolic pathways. The gene distribution is depicted in **(A)** persistent core genes, **(B)** shell genes, and **(C)** cloud genes.

**Table S1:** The complete dataset comprising 75 genomes, including information on genome size, GC content, genome coverage, and isolation site.

**Table S2:** The Excel sheet providing detailed UniProt entries and descriptions of 256 exact core gene families.

**Table S3:** Categorizing the 256 exact core genes into 31 distinct categories with their annotation, number of organisms, and number of sequences.

**Table S4:** A comprehensive overview of the detailed FASTA sequences, and corresponding scores associated with the annotation of the hypothetical proteins.
